# Anti-inflammatory and tissue repair effect of cinnamaldehyde and nano cinnamaldehyde on gingival fibroblasts and macrophages

**DOI:** 10.1186/s12903-023-03682-9

**Published:** 2023-12-19

**Authors:** Mostafa Ghardashpour, Majid Saeedi, Reza Negarandeh, Seyed Ehsan Enderami, Anahita Ghorbani, Anahita Lotfizadeh, Ali Jafari, Alireza Arezoumandi, Hadi Hassannia, Tahereh Molania

**Affiliations:** 1 Dentist, Rasht, Gilan Iran; 2 Department of Pharmaceutics, Faculty of Pharmacy, Mazandaran University of Medical Sciences, Sari, Iran; 3 Student Research Committee, Department of Pharmaceutics, Faculty of Pharmacy, Mazandaran University of Medical Sciences, Sari, Iran; 4 Immunogenetics Research Center, Department of Medical Biotechnology, Mazandaran University of Medical Sciences, Sari, Iran; 5 Department of Oral and Maxillofacial Medicine, Dental Research Center, Faculty of Dentistry, Mazandaran University of Medical Sciences, Sari, Iran; 6 Dentist, Sari, Mazandaran Iran; 7 Department of Paramedicine, Amol School of Paramedical Sciences, Mazandaran University of Medical Sciences, Sari, Iran

**Keywords:** Aphthous stomatitis, Cinnamaldehyde, Herbal medicine, Nano particle, Tissue repair, Treatment

## Abstract

**Background:**

Recurrent aphthous stomatitis has a complex and inflammatory origin. Among the great variety of medications it is increasingly common to use herbal medicines due to the adverse side effects of chemical medications. Considering the anti-inflammatory properties of cinnamaldehyde and the lack of studies related to the effectiveness of its nano form; This study investigates the effect of cinnamaldehyde and nano cinnamaldehyde on the healing rate of recurrent aphthous stomatitis lesions.

**Methods:**

In a laboratory experiment, cinnamaldehyde was converted into niosomal nanoparticles. The niosome vesicles diameter and polydispersity index were measured at 25°C using a dynamic light scattering (DLS) Mastersizer 2000 (Malvern Panalytical technologies: UK) and Zetasizer Nano ZS system (Malvern Instruments Worcestershire: UK). After characterizing these particles, the (2,3-Bis-(2-Methoxy-4-Nitro-5-Sulfophenyl)-2H-Tetrazolium-5-Carboxanilide) [XTT] assay was used to assess the toxicity of cinnamaldehyde and nano cinnamaldehyde on gingival fibroblast (HGF) and macrophage (THP-1) cells. By determining the release of TNF-α, IL-6, and TGF-β cytokines using ELISA kits, the level of tissue repair and anti-inflammatory capabilities of these two substances were evaluated.

**Results:**

The size and loading rate of the cinnamaldehyde nanoparticles were established after its creation. The optimized nanovesicle exhibited the following characteristics: particle size of 228.75 ± 2.38 nm, PDI of 0.244 ± 0.01, the zeta potential of -10.87 ± 1.09 mV and the drug encapsulation percentage of 66.72 ± 3.93%. PDIs range was between 0.242–0.274. The zeta potential values at 25°C were from -2.67 to -12.9 mV. The results of the XTT test demonstrated that nano cinnamaldehyde exhibited dose-dependent toxicity effects. Moreover, nano cinnamaldehyde released more TGF-β and had better reparative effects when taken at lower concentrations than cinnamaldehyde.

**Conclusion:**

Nano cinnamaldehyde and cinnamaldehyde are effective in repairing tissue when used in non-toxic amounts. After confirmation in animal models, it is envisaged that these substances can be utilized to treat recurrent aphthous stomatitis.

## Background

One of the most prevalent conditions affecting the oral mucosa is recurrent aphthous stomatitis.Three kinds of the condition are distinguished clinically: minor, major, and herpetic form [1]. Minor aphthous lesions are less than 5 mm in size, circular, have a definite border, are painful, and heal without leaving scars in ten to fourteen days [2]. The occurrence of this condition is influenced by genetics, bacterial and viral infections, food allergies, vitamin deficiencies, systemic diseases, hormone imbalances, mechanical trauma, and stress [3]. Aphthous lesions can start the immune system's inflammatory responses [4]. Cytokines released from macrophages that have migrated to the wound area, are crucial components in the healing of aphthous lesions [5]. Also, fibroblasts play a critical role in producing new collagen and extracellular matrix structures to support cells involved in repair [6].

Aphthous lesions are being treated with a wide variety of medications. These medications include antiseptics (chlorhexidine), anti-inflammatories (amlexanox), antibiotics (tetracycline), and corticosteroids (triamcinolone acetonide). Systemic treatment with corticosteroids (prednisone) or thalidomide is required when chronic aphthous ulcers develop [7].

Cinnamaldehyde is one of the most beneficial natural remedies for curing aphthous lesions. This chemical, one of the principal ingredients in cinnamon, gives the plant its distinctive aroma [8]. Cinnamaldehyde is a substance with anti-inflammatory, antimicrobial, antioxidant, anti-tumor, anti-diabetic, and nervous system protective properties that can treat many diseases and inflammatory lesions [9].

By limiting the release of inflammatory mediators from mast cells, this chemical lessens the inflammation brought on by aphthous ulcers [10]. Due to its powerful anti-inflammatory characteristics, studies have demonstrated that cinnamaldehyde is utilized in the treatment of osteoarthritis by inhibiting the TLR4/MyD88 pathway in fibroblast cells [11].

This chemical improves gastrointestinal disorders brought on by Helicobacter pylori by blocking the activation pathway (NF-κB), which stops the generation of IL-8 and therefore exerts its anti-inflammatory characteristics [12]. Moreover, this drug greatly lowers the production of nitric oxide, IL-1, IL-6, and TNF-α via influencing macrophages, which are all inflammatory agents [13]. By the stimulation of the PI3K/AKT and MAPK (Mitogen-Activated protein kinase) signaling pathways, this substance also accelerates tissue repair by promoting angiogenesis [14].

Nanotechnology and nano drugs have been used in medicine for various purposes including antifungal, anticancer, and antimicrobial therapies [15]. The size of particles in nanotechnology ranges from 1 to 100 nm. Particles of nanoscale size and modifications to their physical and chemical properties have various benefits and improve the efficiency and effectiveness of medical treatments [16]. Many nanostructures have been studied and employed in dentistry and medicine, including nanometals, nanorobots, nanospheres, nanofibers, and nanorods; These substances enter the body of a person through various ways, and by influencing their DNA or wound healing process, they enhance their systemic and oral health [17, 18]. Studies have shown that Triamcinolone nano-particles and 1% Curcumin nanomicelle gel can be effectively used to enhance the healing of oral aphthous lesions [19, 20]. Moreover, Amlexanox-loaded nanoliposomes have shown a great potential in the treatment of lesions by managing inflammatory condition of oral mucosa. Considering the great efficacy of nanoparticles [21]. The purpose of this study is to produce nano cinnamaldehyde using nanoscience and using other tools and facilities to make this substance better and with better quality to have as much influence as possible on inflammatory lesions such as aphthous lesions and, as a result, increase the speed of recovery of these lesions. Also, in this study, the comparison of cinnamaldehyde and nano cinnamaldehyde will be discussed in terms of their effectiveness and their comparison in different concentrations.

## Methods

In this experimental and laboratory study, the toxicity level of cinnamaldehyde and nano cinnamaldehyde was determined, and their anti-inflammatory and tissue repair efficiency was investigated on gingival fibroblast cell lines and THP-1 macrophages in a cell culture medium. Initially, several cinnamaldehyde formulations were prepared during the steps described below.

### Materials

Cinnamaldehyde (Cinn.), Span 60, tween 60, and cholesterol (Chol.) were purchased from Sigma Aldrich, Samchun (Pure Chemical Co., Ltd. Korea), Sharlua (Sharlab S.L., Spain), and Merck (pharmaceutical Co., Germany), respectively. To purify distilled water, a Human Power 1 device (human Co., Korea) was used.

### Fabrication of niosome of cinnamaldehyde (Cinnosome)

Modified ethanol injection processing was used to prepare the Cinnosome [22]. At first, cholesterol with various amounts, span 60, tween 60 (oily phase), and ethanol were mixed in a beaker by magnetic stirring (150 rpm) at 40–45 °C (detail of the composition for each Cinnosome formulation is listed in Table 1) until dissolved completely. Then, cinnamaldehyde was added to the oily phase. In another beaker, the aqueous phase (water), which had been heated on a hot plate until it reached to a same temperature compare to the oily phase. After that, the oily phase was injected by a syringe into the aqueous phase to create a niosome vesicles by using a hot plate magnetic stirrer. Finally, a probe sonicator (Bandelin;3100; Germany, with a 20% amplitude for 10 min by 45 s rest and 15 s sonication per minute) was used to sonicate the niosome vesicles to produce Cinnamaldehyde-loaded niosomes smaller before freezing the mixture in an ice bath (Table 1).

**Table 1:** Ingredients and Cinnosome characteristics. The data consists of the mean and standard deviation of three different classifications

Formulation	Cinn. (µl)	Tween 60 (mg)	Span 60 (mg)	Chol. (mg)	Ethanol (ml)	Water (ml)	Size (nm)	PDI	Zeta P. (mVolt.)	EE (%)
Cinnosome 1	100	100	100	0	5	Up to 15	137.65 ± 13.35	0.242 ± 0.00	-12.9 ± 0.7	60.72 ± 1.15
Cinnosome 2	100	100	100	50	5	Up to 15	228.75 ± 2.38	0.244 ± 0.01	-10.87 ± 1.09	66.72 ± 3.93
Cinnosome 3	100	100	100	100	5	Up to 15	235.63 ± 4.45	0.253 ± 0.00	-9.53 ± 1.37	61.51 ± 4.85
Cinnosome 4	100	100	100	150	5	Up to 15	242.63 ± 8.61	0.242 ± 0.01	-5.32 ± 0.86	48.46 ± 5.72
Cinnosome 5	100	100	100	200	5	Up to 15	297.17 ± 10.26	0.274 ± 0.03	-2.67 ± 1.04	47.18 ± 4.74

### Characterization of noisome vesicle

The niosome vesicles diameter and PDI^1^ were measured at 25°C using a dynamic light scattering (DLS) Mastersizer 2000 (Malvern Panalytical technologies: UK) and Zetasizer Nano ZS system (Malvern Instruments Worcestershire: UK). To determine the noisome vesicle zeta potential, we used Laser Doppler electrophoresis [23]. Three specimens were taken for each formulation (detail of each Cinnosome formulation is listed in Table 1).

### Entrapment efficacy (EE percentage)

By centrifugation technique, we estimated the percentage of encapsulated cinnamaldehyde in the niosomal vesicles. Colloidal Specimens were centrifuged at 19,000 rpm (SIGMA; 3–30 KS: Germany) for 45 min, followed by filtering (pore size 0.22 μm) the supernatant. By UV spectrophotometer (UV Vis JASCO V-630, UK), we calculated the amount of the free Cinn. in the filtered solution [24] (percentage EE of the Cinnosome formulation was listed in Table 1).

$$\mathrm{EE \% }= \left({\mathrm{W}}_{\mathrm{initial}}-{\mathrm{W}}_{\mathrm{free}}\right) / {\mathrm{W}}_{\mathrm{initial}}$$

W_free_ signifies the amount of drug in the supernatant and W_initial_ denotes the amount of drug put into the formulation.

### FE-SEM^2^ analysis

We used FE-SEM analysis to evaluate noisome vesicle morphology. A drop of the sample was placed on a carbon-coated copper grid. The sample was then dried in air and sputter coated with gold to make it conductive. The images were captured using a scanning electron microscope (HITACHI S-4160) at 20 kV and 15,000 X magnification [25].

### ATR-FTIR^3^ spectroscopy

The interaction between cinnamaldehyde and the other excipients was evaluated by using a Cary 630 FTIR spectrophotometer (Agilent Technologies Inc., CA; the United States) with a diamond ATR. Cinnosome powder (freeze-dried formulation), cinnamaldehyde, cholesterol, Tween 60 and Span 60 were exposed to the ATR-FTIR assessments. The ATR-FTIR spectra were reported in the range of 4000 and 400 cm-1 with a resolution of 2 cm-1 at room temperature [23, 26].

### In vitro drug release

Dissolution apparatus II and Immersion cells with acetate cellulose membrane (MWCO 12 kDa) were used to identify release. The samples were placed in the cells; then the cells were sealed with an acetate cellulose membrane [27]. Afterward, the cells were placed in the dissolution medium filled with 500 mL of water: ethanol (80:20) in each of beakers at 37°C. At different time intervals (1, 2, 4, 6, 8 and 24 h), 5 ml of the dissolution medium were removed, filtered with a 0.22 μm filter paper. The amount of cinnamaldehyde was calculated at 288 nm by a UV spectrophotometer. Also, we added 5 ml of water: ethanol (80:20) in each the dissolution medium of beakers after each sampling to hold the volume of the dissolution medium.

### Cell culture

In this study cell lines were obtained from Pasteur Institute of Iran.

Cell culture takes place in three stages: 1- preparation of culture medium, cell culture and passaging 2- preparation of freeze 3- Thawing.

### Preparation of culture medium, cell culture, and passaging

This study used RPMI 1640 containing 10% FBS. Cells were first counted, and their viability was checked by staining with trypan blue 0.4% diluted in PBS and a hemocytometer slide.

5 × 10^5^ THP-1 cells were cultured in a 25 cm^2^ culture flask containing RPMI culture medium containing 10% FBS and placed inside a cell culture incubator at 37°C and 5% CO_2_.

### Preparation of cell freeze

Consecutive cell freezes must be prepared to store and use the desired cells for a more extended period. At various phases of sub-cloning, THP-1 cells must also be prepared and kept frozen. The freezing atmosphere ought to be richer than others. Additionally, the primary component of freezing, dimethyl sulfoxide (DMSO), is used to stop crystals from forming and damaging cell membranes. To achieve this, an appropriate freezing medium is made using DMSO and FBS in a ratio of nine to one. A suitable cold chain is required for the freezing procedure.

### Thawing

In contrast to the freezing process, where the temperature of the cells should be reduced gradually, the thawing process should be completed rapidly to prevent harm to the cells from DMSO.

### Investigating the cytotoxic effect of cinnamaldehyde and nano cinnamaldehyde on fibroblast and macrophage cell lines in vitro

In order to select the non-toxic concentrations of cinnamaldehyde and nano cinnamaldehyde, fibroblast and macrophage cell lines were treated with different concentrations of cinnamaldehyde and nano cinnamaldehyde (0–50 μM/ml) and the results were evaluated as growth inhibition or non-inhibition by XTT method.

Most papers examined the toxicity of cinnamaldehyde using the MTT method. The MTT test is a colorimetric procedure based on the creation of insoluble blue crystals and the regeneration and breaking of yellow tetrazolium crystals by the enzyme succinate dehydrogenase (one of the enzymes of the mitochondrial respiratory cycle). The benefits of the XTT exam over the MTT include greater accuracy, more uncomplicated application, and time savings. Both adherent and suspended cells are subjected to this assay.

### Measurement of IL-6 and TNF-α cytokines secreted from THP-1 cell line macrophage stimulated with bacterial lipopolysaccharide and treated with cinnamaldehyde and nano cinnamaldehyde by ELISA method

To perform this test, 4 × 10^4^ THP-1 cells were added to each well in a 96-well plate and cultured with bacterial lipopolysaccharide at a concentration of 1 μg/ml for 24 h. After 48 h, the supernatant was collected by centrifuging at a speed of 1000 rpm for 8 min at 4°C then, transferred to a fridge at -20°C.

According to the manufacturer's instructions, IL-6 and TNF-α concentrations were determined using ELISA kits 88–7046 and 88–7346, respectively [28, 29]. PBS and LPS (with a final concentration of 1 μg/ml) were used as negative and positive controls, respectively.

### Measuring TGF-β cytokines secreted from gingival fibroblast cells treated with cinnamaldehyde and nano cinnamaldehyde by ELISA method

8 × 10^3^ HGF cells were added to each well in a 96-well plate. After 48 h, the supernatant was collected by centrifuging at a speed of 1500 rpm for 10 min at a temperature of 4°C and transferred to a fridge at -20°C. TGF-β concentration was determined using an ELISA kit (ab108912) according to the kit manufacturer's instructions.

### Statistical analysis

The statistical study was performed using SPSS 22 and GraphPad Prism 6.01. The Kolmogorov–Smirnov test was used to identify whether the data had a parametric (normal) or non-parametric (non-normal) distribution. The mean and standard deviation for each of statistics is displayed. The Mann–Whitney U-test was employed if the data were non-parametric, and the Student's t-test was applied if the data were normal. The *p*-value was ****p > 0.0001, ***p > 0.001, **p > 0.01, *p > 0.05, and was deemed statistically significant when the level of significance was less than 0.05 with a 95% confidence level. A total of three copies of each experiment were run. It was verified using the Spearman correlation test.

## Results

### Characterization of niosome

We evaluated the hydrodynamic diameter and PDI of NPs^4^ by DLS till affirmed production of nanovesicles and particle size distribution width quality. The range of PDI was between zero to one, when it is closer to zero, it means high uniform dispersion quality [27]. The absolute value of the zeta potential impresses the nanovesicle stability. Various amounts of cholesterol were evaluated (0, 0.25, 0.5, 0.75, 1 w/w) for optimizing the niosomal formulations of cinnamaldehyde. Table 1 presents the niosome vesicle ingredients and their features.

The optimized nanovesicle (Cinnosome 2) was prepared by Ethanol injection technique and exhibited the following characteristics: particle size of 228.75 ± 2.38 nm, PDI of 0.244 ± 0.01, the zeta potential of -10.87 ± 1.09 mV and the drug encapsulation percentage of 66.72 ± 3.93%.

The vesicle diameter increased by increasing the quantity of cholesterol (*P* < 0.05).

PDIs range was between 0.242–0.274 (Table 1) which indicates noisome vesicles were relatively homogeneous in size.

The zeta potential values at 25°C were from -2.67 to -12.9 mV (Table 1), showing that a repulsive electrostatic force exists among the vesicles, which results in stable formulations. In our study, by increasing the amount of cholesterol from 0 to 1%w/w in formulations, the absolute zeta potential of noisome vesicles decreased which was significant (*P* < 0.05).

The percentage of encapsulated cinnamaldehyde in all formulations varied from 47.17 to 66.72 (Table 1). When the amount of cholesterol was 50 mg (0.25%w/w) in Cinnosome 2, the highest drug encapsulation efficiency occurred compared to the other formulations.

When the concentration of cholesterol increased from 0.25 to 1%w/w, encapsulation efficacy was decreased significantly (*P* = 0.002). However, when the amount of cholesterol increased from zero to 50 mg (0.25%w/w) in formulation, the percentage of encapsulation was increased, but that wasn’t significant (*P* = 0.485).

The log p of cinnamaldehyde is 1.9 (lipophilic molecule), and lipophilic molecule can be placed in the hydrophobic bilayers. In the other hands, cholesterol is lipophilic substance and located in the similar situation. So when the amount of cholesterol increases into the mixture, competition substitution on hydrophobic bilayers will increase and reduce cinnamaldehyde encapsulation efficiency in the formulations [30]. All of the characterization examinations were carried out for Cinnosome 2 formulation only.

### FE-SEM analysis

**Fig. 1:**
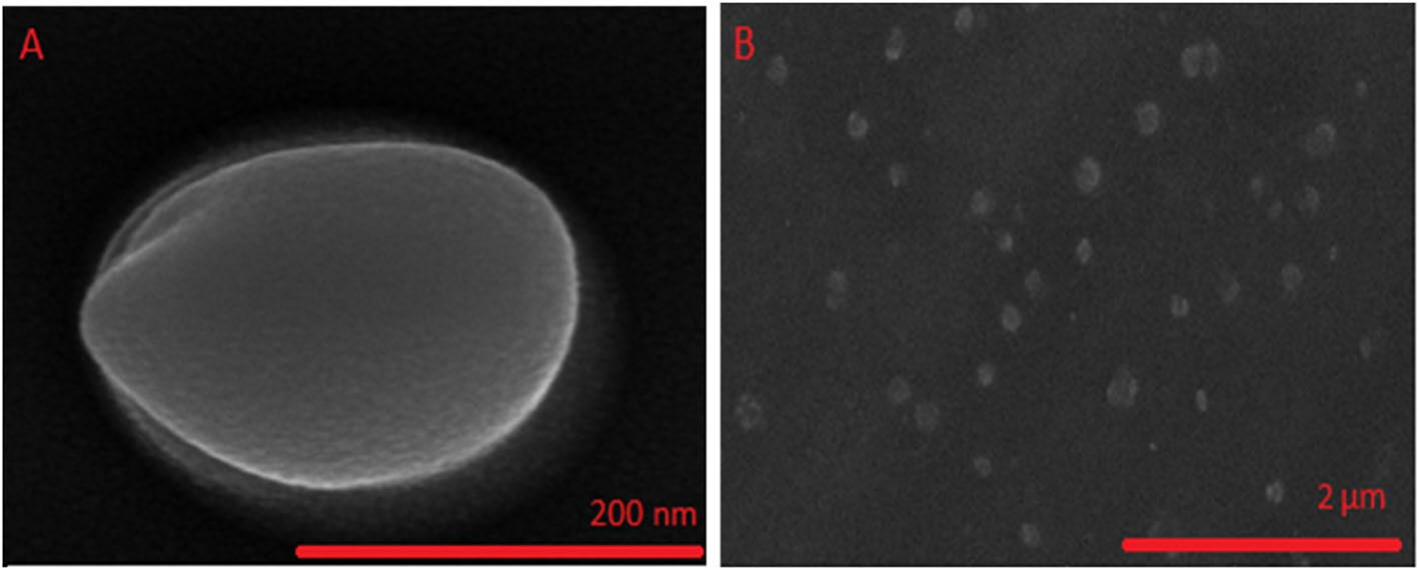
Morphology of Cinnosome 2

To approve the diameter of the vesicle, diameter and surface feature can be determined by this method. As shown, the ideal Cinnosome has a semi-spherical form (Fig. 1.A) and is well-sporadic in size (Fig. 1.B). These findings are consistent with those obtained from the DLS approach and presented in the previous sections of this research.

### ATR-FTIR spectroscopy

**Fig. 2:**
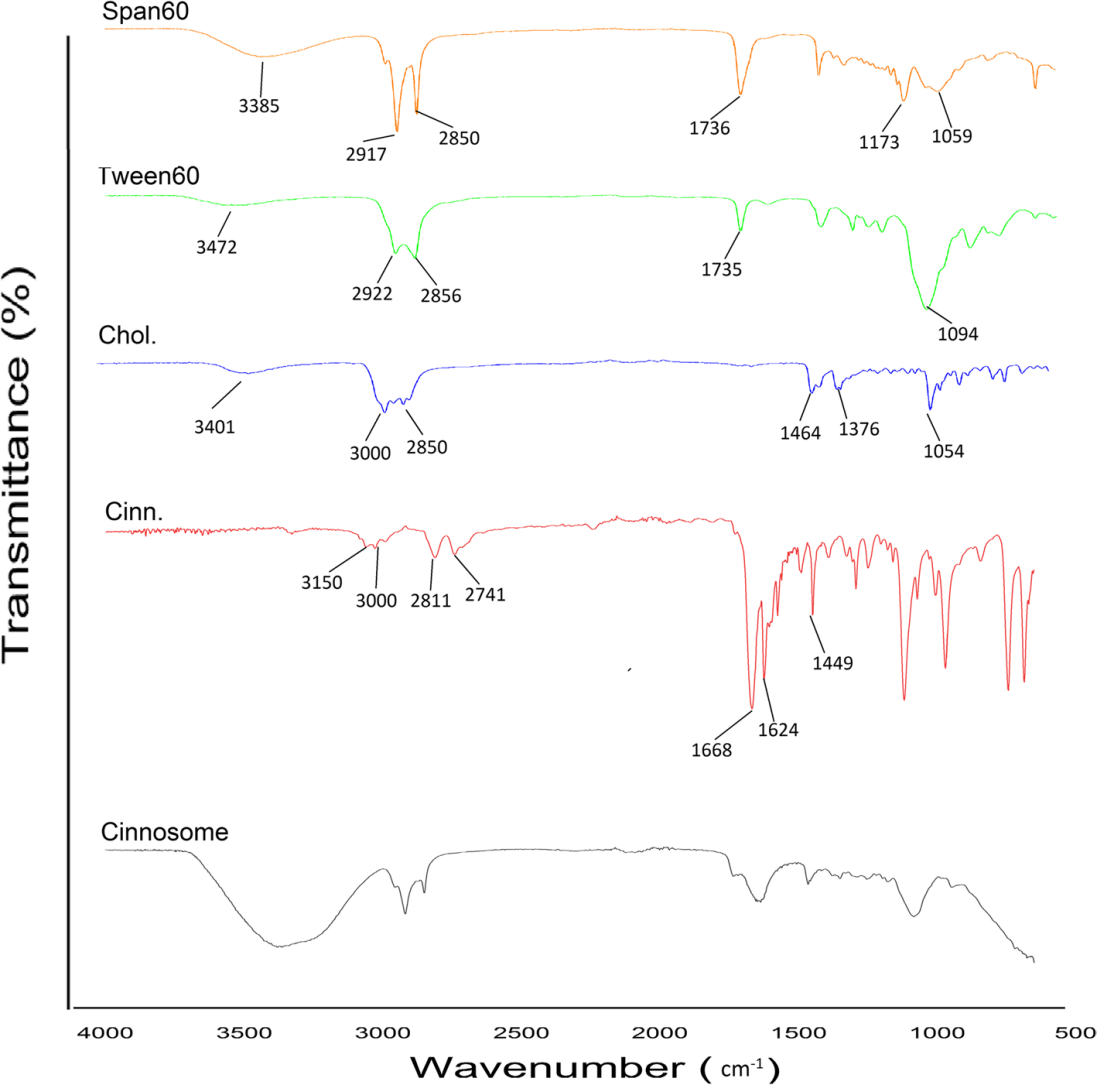
ATR-FTIR spectra of Cinnosome 2, Cinn., Chol, Tween60, Span60

The ATR-FTIR spectra of pure cinnamaldehyde, cholesterol, Tween 60, Span 60, and freeze-dried niosome powder were shown in Fig. 2. The ATR-FTIR spectrum of pure cinnamaldehyde showed the characteristic peaks at 3150–3000 cm^−1^ (aromatic C-H stretching & alkenyl C-H stretching), 2811 cm^−1^ & 2741 cm^−1^ (C-H of aldehyde group stretching), 1668 cm^−1^ (C = O stretching), and 1624–1449 cm^−1^ (alkenyl C = C stretching & aromatic C = C stretching). The spectrum of cholesterol displayed characteristic peaks at 3401 cm^−1^ (O–H stretching), 3000–2850 cm^−1^ (C-H of CH_2_ and CH_3_ groups, asymmetric and symmetric stretching), 1464–1376 cm^−1^ (C-H bending), and 1054 cm^−1^ (C-O stretching). Tween 60 represented peaks at 3472 cm^−1^ (O–H stretching), 2922 cm^−1^ (C-H asymmetric stretching), 2856 cm^−1^ (C-H symmetric stretching), 1735 cm^−1^ (C = O stretching), and 1094 cm^−1^ (C-O stretching). Span 60 demonstrated peaks at 3385 cm^−1^ (O–H stretching), 2917 cm^−1^ (C-H asymmetric stretching), 2850 cm^−1^ (C-H symmetric stretching), 1736 cm^−1^ (C = O stretching), and 1173 cm^−1^ & 1059 cm^−1^ (C-O stretching).

As shown by the ATR-FTIR results, there was no chemical interaction among cinnamaldehyde and the other excipients in the selected formulation. Also, the characteristic peaks of pure cinnamaldehyde are remained in the Cinnosome 2 powder, which indicate that cinnamaldehyde is entrapped in noisome vesicle without chemical interaction among the other excipients.

### In vitro drug release

**Fig. 3:**
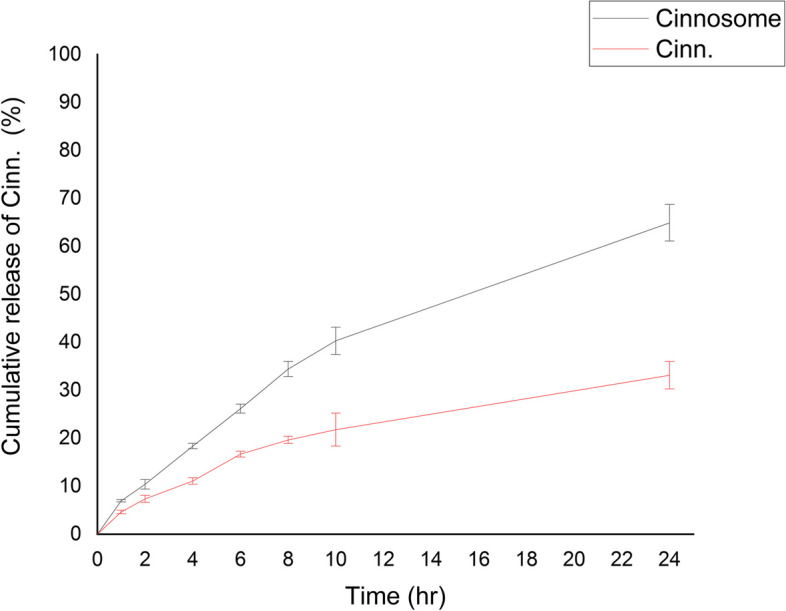
Dissolution profile of plain cinnamaldehyde solution and Cinnosome 2 formulation

The in vitro drug release profile of Cinnosome 2 and pure cinnamaldehyde is shown in Fig. 3. For the Cinnosome formulation, the first-order release model provided the greatest fit (R^2^ = 0.992). The other kinetic models, such as Korsmeyer-Peppas, Higuchi, Hixon-Crowell, and zero, had R^2^ values of 0.991, 0.976,0.586, and 0.94, respectively. The findings showed that after 24 h, cinnamaldehyde release from the Cinnosome formulation (64.91 ± 3.81) was faster than the drug release from the pure cinnamaldehyde formulation (33.14 ± 2.87) (*P* < 0.001).

### The results of investigating the effect of cytotoxicity of cinnamaldehyde and nano cinnamaldehyde on HGF fibroblast cell line in vitro using XTT test

**Fig. 4:**
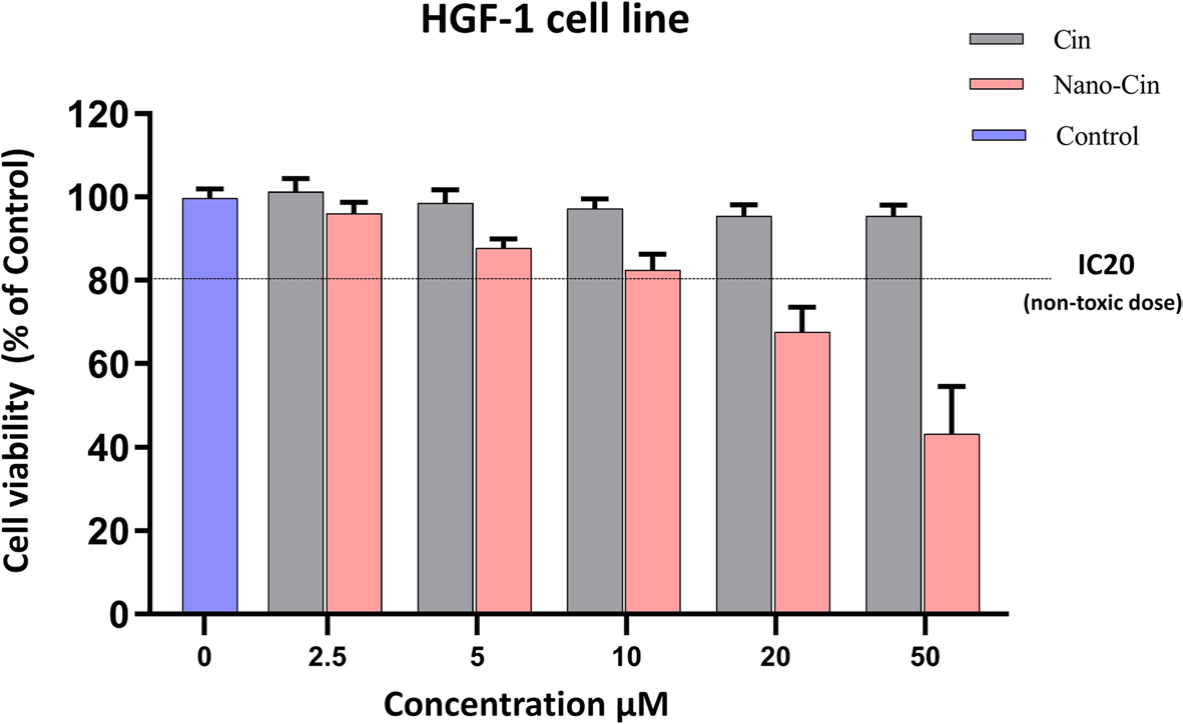
The results of investigating the toxicity effect of different concentrations of cinnamaldehyde and nanocinnamaldehyde on the HGF fibroblast cell line

Figure 4 shows the effect of different concentrations of these substances (2.5, 5, 10, 20 and 50 μM) after 48 h of cell exposure. The results showed that cinnamaldehyde had no toxicity in 48 h at different concentrations. In contrast, nano cinnamaldehyde showed its toxic effects on HGF cells in a dose-dependent manner. Therefore, IC20 was used to select a non-toxic dose (11.8 μM) in order to further investigate its impact on the function of these cells.

### The results of investigating the effect of cytotoxicity of cinnamaldehyde and nano cinnamaldehyde on THP-1 macrophage cell line in vitro using XTT test

**Fig. 5:**
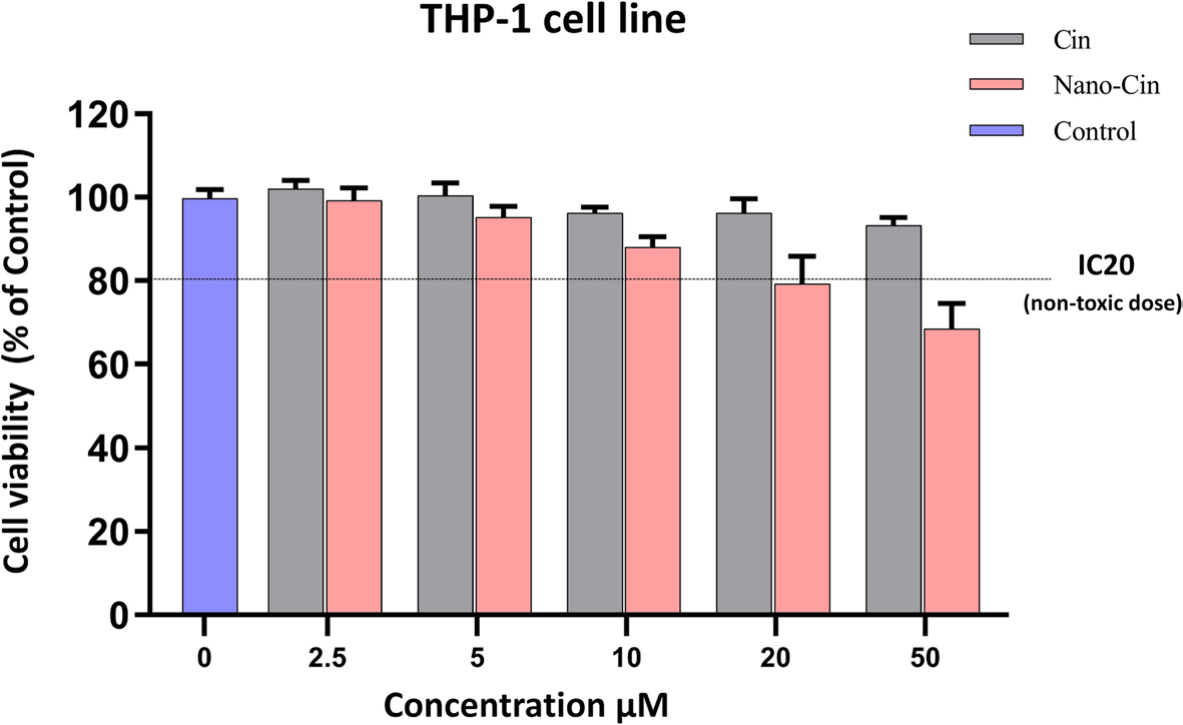
The graph of the results of investigating the toxicity effect of different concentrations of cinnamaldehyde and nano cinnamaldehyde on the THP-1 macrophage cell line

Figure 5 shows the effect of different concentrations of these substances (2.5, 5, 10, 20, and 50 μM) after 48 h of cell exposure. The results showed that cinnamaldehyde has no toxicity in 48 h at different concentrations, while nano cinnamaldehyde showed dose-dependent toxicity effects on the THP-1 macrophage cells. Therefore, IC20 was used to select a non-toxic dose to investigate the impact of its non-toxic dose (19.7 μM) on the function of these cells.

### The results of measurement of TNF-α cytokine secretion from the THP-1 cell line macrophages treated with cinnamaldehyde and nano cinnamaldehyde by ELISA (Enzyme-linked immunosorbent assay) method

**Fig. 6:**
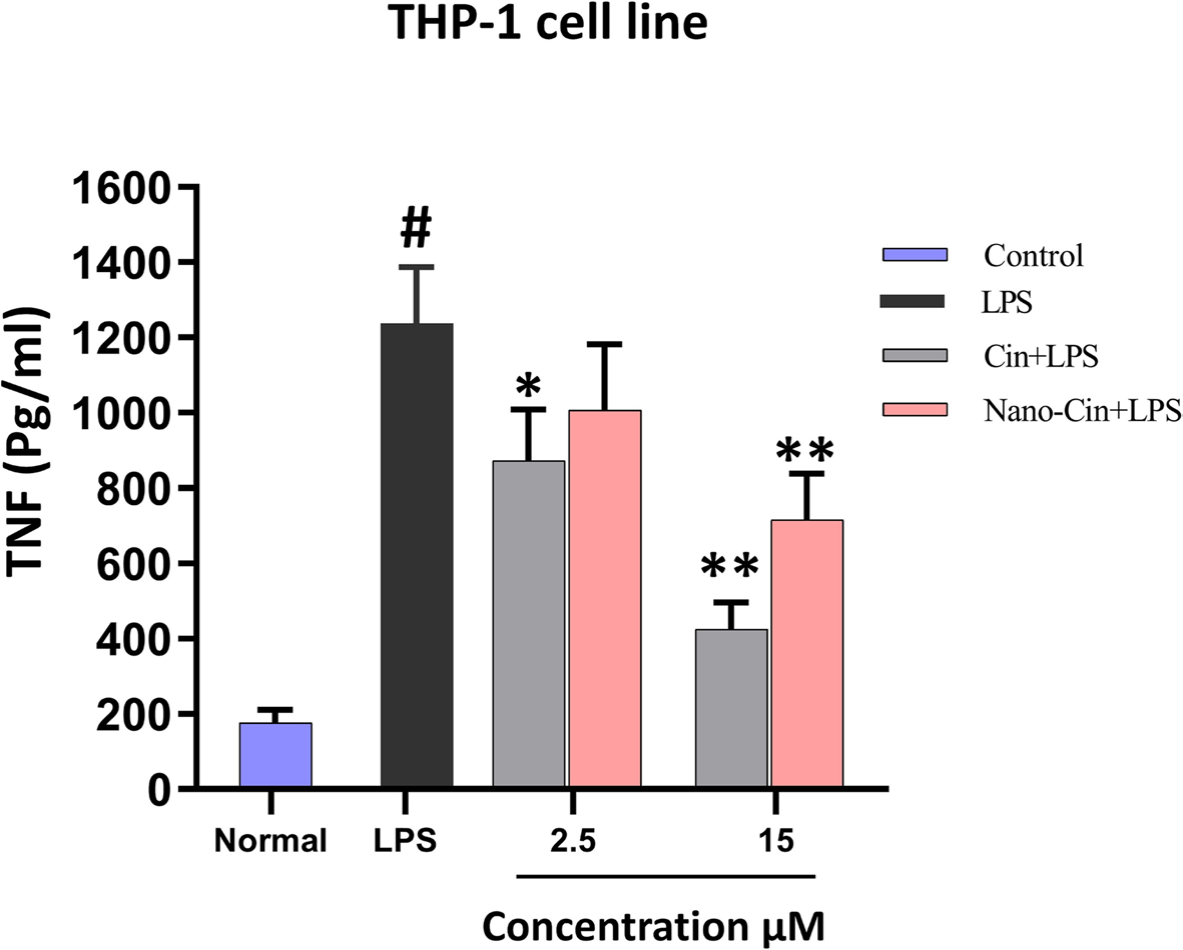
Graph of the results of the effect of non-toxic doses of cinnamaldehyde and nano cinnamaldehyde on TNF cytokine secretion from THP-1 macrophage cells

As shown in Fig. 6, compared to the LPS (lipopolysaccharide) control group (to stimulate TNF-α production), the cinnamaldehyde group in both doses of 2.5 and 15 μM and the nano cinnamaldehyde group only in the dose of 15 μM were able to significantly show their anti-inflammatory effects in reducing the secretion of cytokine TNF-α after stimulation by LPS. Also, the effect of cinnamaldehyde on the secretion of TNF-α cytokine is higher compared to nano cinnamaldehyde and it inhibits the secretion of this inflammatory cytokine to a higher extent. To confirm these results, a statistical comparison of the amount of TNF-α secretion between different groups is given in Table 2.

**Table 2:** Statistical comparison of the level of TNF secretion in the presence of LPS after treatment in different groups

Comparison between two groups		*P*-Value
Cin 2.5 + LPS	LPS	0.0159^*^
Cin 15 + LPS	LPS	0.0079^**^
Nano-Cin 2.5 + LPS	LPS	0.0635
Nano-Cin 15 + LPS	LPS	0.0079^**^

^*^P-value < 0.05

^**^P-value < 0.01

### The results of measurement of IL-6 cytokine secretion from THP-1 cell line macrophages treated with cinnamaldehyde and nano cinnamaldehyde by ELISA method

**Fig. 7:**
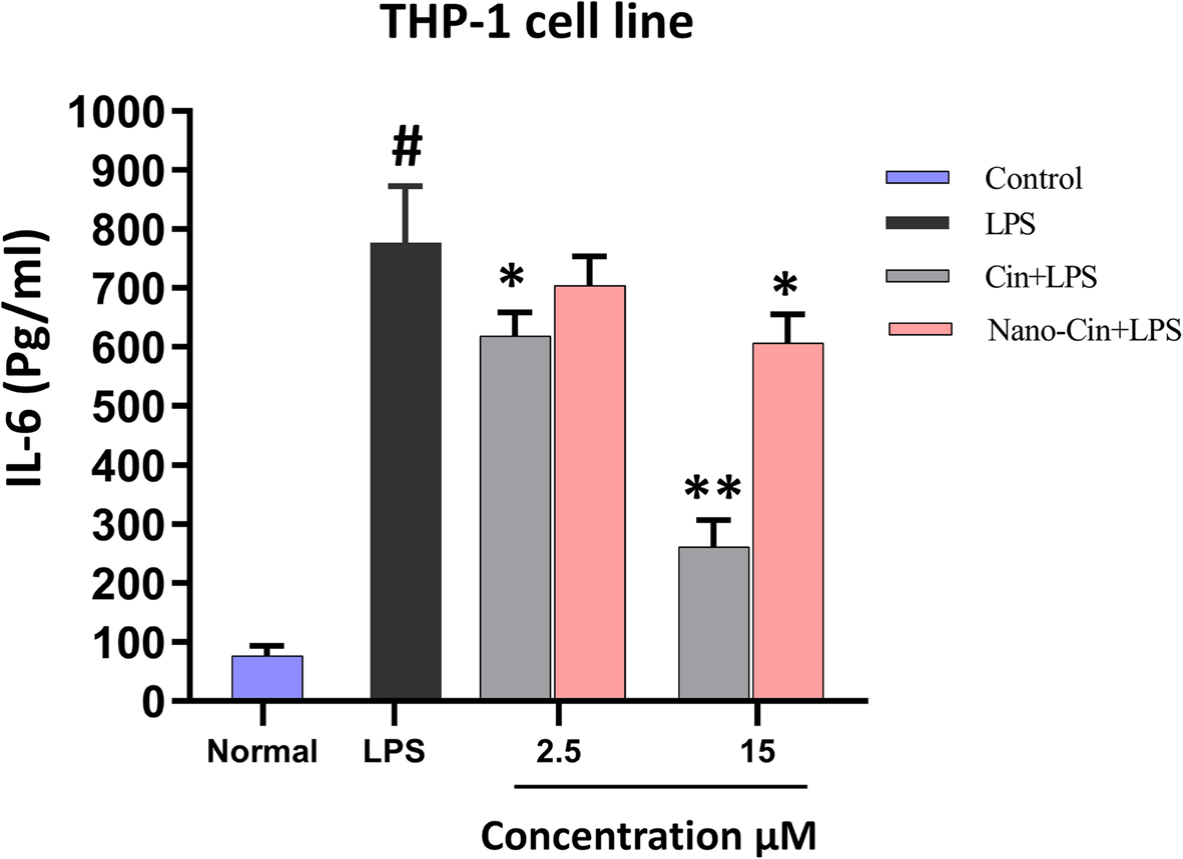
The effect of non-toxic doses of cinnamaldehyde and nanocinnamaldehyde on IL-6 cytokine secretion from THP-1 macrophage cells

As shown in Fig. 7, compared to the LPS control group (to stimulate IL-6 production), the cinnamaldehyde group at both doses of 2.5 and 15 μM and the nano cinnamaldehyde group only at the dose of 15 μM significantly showed their anti-inflammatory effects in reducing IL-6 cytokine secretion after LPS stimulation. Also, the effect of cinnamaldehyde on IL-6 cytokine secretion is higher compared to nanocinnamaldehyde and inhibits the secretion of this inflammatory cytokine to a higher extent. To confirm these results, a statistical comparison between the amount of IL-6 secretion between different groups is given in Table 3.

**Table 3:** Statistical comparison of the level of IL-6 secretion in the presence of LPS after treatment in different groups

Comparison between two groups		*P*-Value
Cin 2.5 + LPS	LPS	0.0159^*^
Cin 15 + LPS	LPS	0.0079^**^
Nano-Cin 2.5 + LPS	LPS	0.1111
Nano-Cin 15 + LPS	LPS	0.0159^*^

^*^P-value < 0.05

^**^P-value < 0.01

### The results of measurement of TGF-β cytokine secretion from gingival fibroblast (HGF) cells treated with cinnamaldehyde and nano cinnamaldehyde by ELISA method

**Fig. 8:**
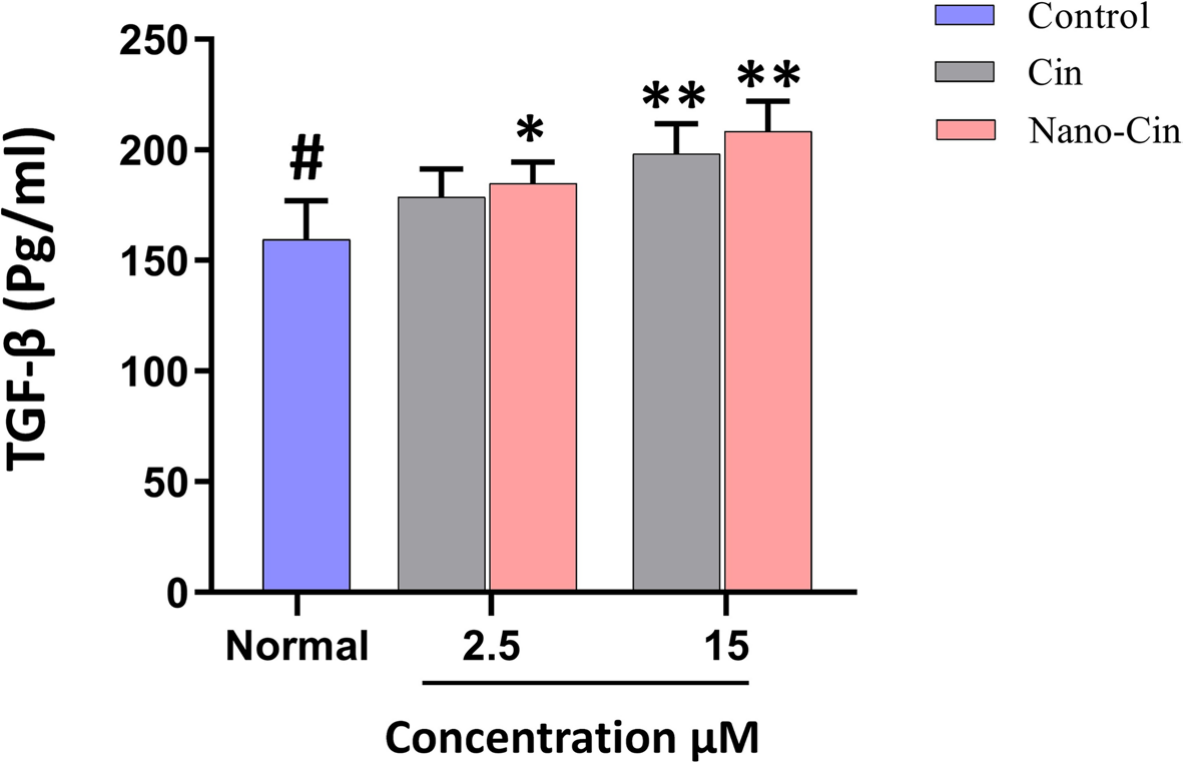
The results of the effect of non-toxic doses of cinnamaldehyde and nano cinnamaldehyde on TGF-β cytokine secretion from HGF cells

The results of the effect of these substances on the HGF cell line in picograms per milliliter was measured by ELISA method; the results are given in Fig. 8. The results show that cinnamaldehyde nanoparticles in low doses (2.5 μM) have stronger effects than cinnamaldehyde in the same dose on the stimulation of TGF-β cytokine secretion. But these effects seem to be saturated in higher doses and do not have a statistically significant difference from each other.

To confirm these results, the statistical comparison between the amount of TGF-β secretion between different groups is given in Table 4.

**Table 4:** Statistical comparison of the amount of TGF-β secretion in the presence of non-toxic doses of cinnamaldehyde and nano cinnamaldehyde after treatment in different groups

Comparison between two groups		*p*-Value
Cin 2.5	Control	0.1667
Cin 15	Control	0.0079^**^
Nano-Cin 2.5	Control	0.0317^*^
Nano-Cin 15	Control	0.0079^**^

^*^P-value < 0.05

^**^P-value < 0.01

## Discussion

### Characterization of noisome

The vesicle diameter increased by increasing the ration of cholesterol/surfactant (*P* < 0.05). In line with the present study, Tajbakhsh et al. found that higher cholesterol contents in noisome vesicles of testosterone enanthate can increase the diameter of niosomes [23]. Tavano et al. evaluated the relationship between the content of cholesterol and the particle size of the diclofenac sodium vesicles; then they observed that by increasing hydrophobicity, smaller vesicles were created [31]. Our result was similar to mention studies.

In our study, by increasing the amount of cholesterol from 0 to 1%w/w in formulations, the absolute zeta potential of noisome vesicles decreased and it was significant (*P* < 0.05). Abootorabi et al. indicated that increasing the cholesterol content in noisome vesicle from 0.25 to 1.25%w/w decreases absolute zeta potential but it wasn’t significant [30]. Also, Tajbakhsh et al. showed that by increasing amount of cholesterol contents in noisome vesicles, absolute zeta potential of vesicles decreased and it was significantly [23].

When the amount of cholesterol was 50 mg (0.25%w/w) in Cinnosome 2, the highest drug encapsulation efficiency occurred compared to the other formulations. Nasseri et al. reported that the highest amount of cholesterol in the formula, enhanced the encapsulation efficiency [32]. Also in studies of Tajbakhsh et al., encapsulation of testosterone enanthate in noisome vesicles increased by increasing amount of cholesterol in formulations [23]. But in the present study, when the concentration of cholesterol increased from 0.25 to 1%w/w, encapsulation efficacy was decreased significantly (*P* = 0.002). However, when the amount of cholesterol increased from zero to 50 mg (0.25%w/w) in formulation, the percentage of encapsulation was increased, but that wasn’t significant (*P* = 0.485). the reason of this subject may occurred competition of cholesterol with cinnamaldehyde for substitution in bilayer of noisome vesicles.

### Determination of cytotoxicity of cinnamaldehyde and nano cinnamaldehyde

In the present study, the effect of these substances on the survival rate of gingival fibroblast and THP-1 macrophage cell lines was examined using the XTT method to select non-toxic concentrations of cinnamaldehyde and nano cinnamaldehyde. Results revealed that cinnamaldehyde had no toxicity in 48 h at various concentrations after the effect of different concentrations of cinnamaldehyde and nano cinnamaldehyde (2.5, 5, 10, 20, and 50 μM) during cell exposure, whereas nano cinnamaldehyde displayed dose-dependent deleterious effects. We observed a greater level of cytotoxicity from nano cinnamaldehyde compared to cinnamaldehyde because the drug's nano form increased cell uptake and degree of drug penetration into the cell.

Therefore, to select a non-toxic dose, IC20 was used to investigate the effect of a non-toxic dose on THP-1 macrophage, gingival fibroblast cells (19.7 and 11.8 μM, respectively), and the function of these cells. The IC20 dose is the concentration at which the growth of 20% of the cells is inhibited. This dose elicits a response in the cell without being excessively toxic [33], and allows the identification of subtle and compound-specific biological effects [34]. In a laboratory experiment examining the impact of kidney cancer-causing bimetallic titanocene-gold phosphane, Benelita Elie et al. also employed the IC20 dose of this drug [35]. Furthermore, the IC20 dose of ruthenium was utilized in a laboratory investigation by Mohamed Kasim et al. about the toxicity and antimetastatic action of ruthenium because greater levels caused cells to be highly toxic and deadly [36].

The protective role of cinnamaldehyde against oxidative stress in human dental pulp cells was also studied in a laboratory study by Nam-Yi Kim et al. Dental pulp cells were exposed to 1 to 50 μM of cinnamaldehyde for 24 h to test for toxicity, and the MTT test was performed to assess cell viability. According to this investigation, cinnamaldehyde did not exhibit any discernible harmful effects up to a concentration of 50 μM [37]. In accordance with the findings of the current research, Nguyet Tran Trinh et al. investigated the protective effect of cinnamaldehyde in the presence of oxidative stress and its inhibitory effect on the secretion of TNF-α in the inflammatory responses of human umbilical vein endothelial cells. Cinnamaldehyde in concentrations ranging from 1 to 80 M was applied to endothelial cells for 24 h to assess its toxicity. According to the findings of this study, cinnamaldehyde does not significantly harm human umbilical vein endothelial cells up to a concentration of 40 μM. However, at higher concentrations, it exhibited a dose-dependent toxic effect [38]. In a lab experiment to improve transfection efficiency in retinal pigment epithelial cells, Yameni Qin et al. investigated the effect of cationic hyaluronic acid niosomes. In this study, niosomes were produced by an ethanol injection method and then hyaluronic acid was added to them. 0.5 to 2.5 μM of niosome concentrations were utilized for 24 h in the MTT test to investigate toxicity. The findings demonstrated that nanoparticles did not exhibit a substantial toxicity effect up to a concentration of 2 μM, but at higher concentrations, the toxicity effect significantly increased [39]. The possible toxic impact of curcumin niosomes on skin fibroblast cells was additionally examined in a different laboratory investigation by Akbari et al.; using the MTT test and niosome material concentrations ranging from 1.5 to 15 mg/ml for 24 h. The findings indicated none of the employed amounts had a detectable harmful effect [40]. The inhibitory effect of acyclovir micro nisomes on herpes simplex virus type 1 in cell culture medium was explored in a laboratory investigation by Monavari et al.; HeLa cells and the MTT test were used to determine the toxicity of the nano drug in this study. The nano-drug form was created using the ethanol injection technique. Amounts of 5 to 30 µM of the nano-drug form were used on uninfected HeLa cells for 48 h. The findings demonstrated that at concentrations of 5 to 15 μM, acyclovir nanonisome had no detectable toxicity effect, but at higher concentrations, it exhibited dose-dependent toxicity effects [41]. Moreover, Mahmood Barani et al. investigated the impact of niosomes containing thymoquinone on breast cancer cells in another laboratory experiment. Nisome nanoparticle concentrations ranging from 0.5 to 10 μM were employed in this investigation on cancer cells for 24 h, and toxicity was assessed using the MTT assay. The findings demonstrated that these nanoparticles did not have a significant harmful effect at a concentration of 5 μM, but at a concentration of 10 μM, they had a harmful effect, and cell viability drastically dropped [42].

Somaiya Mateen et al. conducted a laboratory investigation to examine the anti-inflammatory and antioxidant effects of cinnamaldehyde and eugenol on rheumatoid arthritis patients' peripheral blood cells. In this study, PBMC cells were exposed to cinnamaldehyde and eugenol at concentrations ranging from 10 to 40 μM, and the MTT assay was utilized to assess toxicity. The findings demonstrated that these two chemicals did not significantly alter the cell viability rate or have a harmful effect at the quantities used [43]. Similar to the current investigation, Louis Kuoping Chao et al., in a study, examined the inhibition of cinnamaldehyde on inflammatory cytokine release from monocyte and macrophage cells via altering intracellular signaling pathways. In this study, 8 to 80 μM of cinnamaldehyde was used on THP-1 macrophage cells, and the MTT test was used to determine the toxicity level. The results showed that cinnamaldehyde in the amounts used had no significant toxicity effect [8].

### Anti-inflammatory effect of cinnamaldehyde and nano cinnamaldehyde on macrophage cells

After determining the IC-20 and selecting low-toxic doses (2 doses of 2.5 and 15 μM) of cinnamaldehyde and nano cinnamaldehyde, to determine the extent of their anti-inflammatory properties, the effect of cinnamaldehyde and nano cinnamaldehyde on inhibiting the stimulation of the secretion of cytokines TNF-α and IL-6 obtained from THP-1 cell line macrophage was investigated. Compared to the LPS control group (to stimulate the production of TNF-α and IL-6), the cinnamaldehyde group at both doses of 2.5 and 15 μM and the nano cinnamaldehyde group only at a dose of 15 μM were able to significantly show their anti-inflammatory effects in reducing the secretion of TNF-α and IL-6 cytokines when stimulated with LPS. Also, the impact of cinnamaldehyde on the secretion of TNF-α and IL-6 cytokines is higher than nano cinnamaldehyde and inhibits the secretion of these inflammatory cytokines to a greater extent. To confirm these results, a statistical comparison between the amount of TNF-α and IL-6 cytokines secretion between different groups is given in Tables 2 and 3.

Also, in a laboratory study conducted in 2020 by Anmin Ruan et al., the inhibitory effect of cinnamaldehyde on inflammation caused by lipopolysaccharide stimulation in chondrocyte cells and degeneration of cartilage tissue was investigated by blocking the NF-κB signaling pathway. In this study, chondrocyte cells were first treated with 10–50 µM of cinnamaldehyde and then stimulated with 1 µM of bacterial lipopolysaccharide. The results showed that cinnamaldehyde significantly reduced the production of inflammatory cytokines such as TNF-α, IL-6, and IL-1β [44]. Similar to the current research, Hang Zhao et al., in a laboratory experiment on mice with heart dysfunction and stimulated by lipopolysaccharide, examined the anti-inflammatory effects of cinnamaldehyde by influencing the TLR4-NOX4 pathway. In this research, the secretion of the cytokines TNF- α, IL-6, and IL-1 was measured using ELISA kits 4 h after the stimulation of mice with bacterial lipopolysaccharide and treatment with amounts of 30 to 90 mg/kg of cinnamaldehyde. Cinnamaldehyde significantly decreased inflammatory cytokines, according to the findings [45]. In a laboratory study, Jung-Chun et al. investigated the anti-inflammatory effect of the constituents of Cinnamomum cassia (cinnamon). In the mentioned study, RAW264.7 macrophage cells were stimulated with 100 μM of LPS and then treated with 12.5 to 50 μM of cinnamaldehyde. Then, to investigate the anti-inflammatory effect of cinnamaldehyde, the amount of secretion of TNF-α and PGE2 was evaluated used using ELISA kits. The results showed that cinnamaldehyde significantly reduced the secretion of TNF-α and PGE2 (prostaglandin E2) cytokines from macrophage cell [46].

Additionally, Louis Kuoping Chao et al. examined the inhibitory effect of cinnamaldehyde on the secretion of pro-inflammatory cytokines in macrophage and monocyte cells by inhibiting intracellular signals in a laboratory and experimental study carried out in 2007. In this research, j774A and THP-1 macrophage cells were treated with 1 μM of lipopolysaccharide and 8 to 80 μM of cinnamaldehyde. The results demonstrated that the secretion of TNF-α and IL-6 cytokines decreased in J774A.1 macrophage cells. Additionally, treatment with cinnamaldehyde reduced the rate of TNF-α and IL-1 cytokine release in THP-1 macrophage cells [8].

### The effect of cinnamaldehyde and nano cinnamaldehyde on the activity of gingival fibroblasts

Figure 7 shows the results of the effect of cinnamaldehyde and nano cinnamaldehyde on the HGF (human gingival fibroblast) cell line in picograms per milliliter as measured by the ELISA method after examining the toxicity of cinnamaldehyde and nano cinnamaldehyde in the mentioned concentrations and determining the non-toxic concentrations of these two substances and using them to determine the extent of their regenerative properties. The findings demonstrate that cinnamaldehyde nanoparticles at modest doses (2.5 μM) have more potent effects on stimulating TGF-β cytokine secretion than cinnamaldehyde at the same dose. However, these effects do not appear to vary statistically significantly from one another and appear to be saturated at larger doses. To confirm these results, the statistical comparison between the amount of TGF-β secretion between different groups is given in Table 4.

Several processes are involved in the improvement of the tissue during the highly complicated process of wound healing. Four overlapping phases of this process with predictable biochemical and cellular processes are tissue formation (proliferative phase), homeostasis, inflammation, and ultimately tissue regeneration. Numerous elements, including cytokines, growth hormones, and low molecular weight substances, impact these processes [47].

A wide variety of various cell types also take part in the body's response to wound healing. Some crucial cells include mesenchymal progenitor cells, fibroblasts, neutrophils, monocytes and macrophages, lymphocytes, endothelial cells, and keratinocytes [48]. The most essential cell involved in the repair of inflammatory lesions and tissue repair is the fibroblast. These cells are essential in supporting routine wound healing and play a key role in crucial processes such as fibrin clot breakdown, creation of new extracellular matrix (ECM), collagen structures to support other cells involved in effective wound healing, and wound contraction [49].

A group of repair factors and cytokines, in addition to the cells engaged in the healing of wounds and inflammatory lesions, also play a critical role. Transforming growth factor beta (TGF-β), one such substance is released by various cell types, including platelets, activated macrophages, neutrophils, fibroblasts, keratinocytes, endothelial cells, T cells, and others. A protein called TGF-β has a molecular weight of approximately 25 kDa and 112 amino acid monomer units [50]. This growth factor, secreted by fibroblasts, regulates fibroblast proliferation, differentiating them into myofibroblasts, promoting the creation of extracellular matrix, and promoting the synthesis of collagen, elastin, and fibronectin.

## Conclusion

The findings demonstrated that nano cinnamaldehyde compared to cinnamaldehyde, has a more toxic impact on gingival fibroblast cells and THP-1 macrophages at comparable doses. Additionally, cinnamaldehyde has more anti-inflammatory effects than nano cinnamaldehyde while having fewer effects on tissue repair at the same non-toxic levels. Cinnamaldehyde and its nano form can treat recurrent aphthous stomatitis because both substances have anti-inflammatory properties and effectively repair damaged tissue, but this topic still requires more research.

## Data Availability

All data generated or analysed during this study are included in this published article.

## Abbreviations

XTT: (2,3-Bis-(2-Methoxy-4-Nitro-5-Sulfophenyl)-2H-Tetrazolium-5-Carboxanilide)
TNF-α: Tumor necrosis factor-alpha
TGF-β: Transforming growth factor beta
PDGF: Platelet-derived growth factor
EGF: Epidermal growth factor
bFGF: Fibroblast growth factor
IL: Interleukin
MAPK: Mitogen-activated protein kinase
PDI: Polydispersity index
FE-SEM: Field emission scanning electron microscopy
ATR-FTIR: Attenuated total reflectance-fourier transform infrared
NPs: Nanoparticles
LPS: Lipopolysaccharide
ELISA: Enzyme-linked immunosorbent assay
PGE2: Prostaglandin E2
HGF: Human gingival fibroblast

## Footnotes

1 polydispersity index.

2 Field emission scanning electron microscopy.

3 Attenuated total Reflectance-Fourier transform infrared.

4 nanoparticles.
